# Algorithm for real-time analysis of intracoronary electrocardiogram

**DOI:** 10.3389/fcvm.2022.930717

**Published:** 2022-09-07

**Authors:** Marius Reto Bigler, Andrea Kieninger-Gräfitsch, Frédéric Waldmann, Christian Seiler, Reto Wildhaber

**Affiliations:** ^1^Department of Cardiology, University Hospital Bern (Inselspital), University of Bern, Bern, Switzerland; ^2^Institute for Medical Engineering and Medical Informatics, University of Applied Sciences and Arts Northwestern Switzerland, Muttenz, Switzerland; ^3^Signal and Information Processing Laboratory (ISI), ETH Zürich, Zurich, Switzerland

**Keywords:** autonomous algorithm, intracoronary electrocardiogram, myocardial ischaemia, ECG-analysing system, coronary artery disease

## Abstract

**Introduction:**

Since its first implementation in 1985, intracoronary (ic) electrocardiogram (ECG) has shown ample evidence for its diagnostic value given the higher sensitivity for myocardial ischemia detection in comparison to surface ECG. However, a lack of online systems to quantitatively analyze icECG in real-time prevents its routine use. The present study aimed to develop and validate an autonomous icECG analyzing algorithm.

**Materials and methods:**

This is a retrospective observational study in 100 patients with chronic coronary syndrome. From each patient, a non-ischemic as well as ischemic icECG at the end of a 1-min proximal coronary balloon occlusion was available. An ECG expert as well as the newly developed algorithm for autonomous icECG analysis measured the icECG ST-segment shift in mV for each icECG tracing.

**Results:**

Intraclass correlation coefficient (ICC) demonstrated low variability between the two methods (ICC = 0.968). Using the time point of icECG recording as allocation reference for absent or present myocardial ischemia, ROC-analysis for ischemia detection by the manually determined icECG ST-segment shift showed an area under the curve (AUC) of 0.968 ± 0.021 (*p* < 0.0001). AUC for the algorithm analysis was 0.967 ± 0.023 (*p* < 0.0001; *p* = 0.925 for the difference between the ROC curve AUCs). Time to complete analysis was below 1,000 ms for the autonomous icECG analysis and above 5 min for manual analysis.

**Conclusion:**

A newly developed autonomous icECG analysing algorithm detects myocardial ischemia with equal accuracy as manual ST-segment shift assessment. The algorithm provides the technical fundament for an analysing system to quantitatively obtain icECG in real-time.

## Introduction

The electrocardiogram (ECG) is an indispensable tool in the diagnosis of myocardial ischemia. However, the commonly used surface ECG is limited in detecting short-lasting or minor myocardial ischemia, especially in the territory of the left circumflex coronary artery (LCX) (i.e., the posterior wall of the left ventricle) (1, 2). An ECG recorded in close vicinity to the myocardial region of interest has been thought to overcome these spatial limitations. Hence, in 1976, Hashimoto et al. (3) were the first to record an intracoronary ECG (icECG), with electrodes placed within a canine coronary artery. They used the coronary guidewire as an electrode; thus, the recorded (pseudo)unipolar icECG reflected the local epicardial ECG. Using this setup, the authors analyzed the course of the icECG ST-segment shift during complete coronary balloon occlusion in 18 dogs for up to 3 h (3). Meier and Rutishauser later employed icECG during percutaneous transluminal coronary angioplasty (PTCA) in men (4). Since then, icECG has been shown to be of diagnostic value given the higher sensitivity for myocardial ischemia detection when compared to surface ECG. In this context, it has been proven accurate to guide percutaneous coronary interventions and to predict post-procedural myocardial injury (5–9). However, the lack of an automatic analyzing system to quantitatively assess icECG in real-time has prevented routine use (10), in particular, as current manual analysis requires cumbersome data preparation, conversion and analysis on customized software resulting in a time-consuming process of 5–10 min per patient.

Thus, the goal of this study was to develop and validate an autonomous intracoronary ECG analyzing algorithm. In a first step, an algorithm for quantitative assessment of ST-segment shift (in mV) was developed. The present study tested the accuracy of the algorithm in comparison to manual quantitative analysis of icECG ST-segment shift for the detection of artificially induced myocardial ischemia.

## Materials and methods

### Study design and patients

**FIGURE 1:**
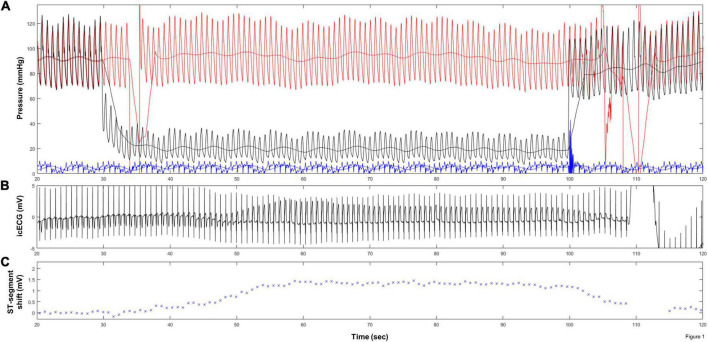
Collateral flow index (CFI) measurement. **(A)** Simultaneous recordings of mean and phasic aortic (red signals), coronary occlusive (black signals), central venous pressure (blue signals), and intracoronary electrocardiogram (ECG) **(B)** immediately before (left side) and during coronary artery occlusion in a patient with poorly functional collaterals. **(C)** Quantitative measurement at the ST-segment shift for every single heartbeat.

This is a retrospective observational study in 100 patients with chronic coronary syndrome undergoing coronary angiography due to chest pain, who participated in clinical trials of our research group with determination of coronary collateral flow index (CFI), the quantitative measure of coronary collateral function during a brief, artificial coronary occlusion (11, 12). A detailed description of CFI has been previously published (13). In brief, CFI is a measure of coronary collateral blood supply obtained during a 1-min proximal coronary artery balloon occlusion defined as mean coronary occlusive pressure relative to mean aortic pressure, both subtracted by central venous pressure (14) (Figure 1A). In the absence of sufficient collateral blood supply, coronary balloon occlusion induces maximal myocardial ischemia at the end of the occlusion. Using this experimental setting of a brief artificial coronary balloon occlusion allowed an independent reference essential for the required statistical analysis. Hence, the present study employed the same temporal landmarks with non-ischemic (i.e., before coronary occlusion), and controlled ischemic (i.e., at the end of the occlusion) conditions as previously described (15). Criteria for patient inclusion in this retrospective study were measurement of CFI with simultaneous recording of icECG (Figures 1A,B), and written informed consent for further use of the patient’s data. Exclusion criteria were prior Q-wave myocardial infarction in the vascular territory undergoing icECG measurement, presence of bundle branch blocks, non-sinus rhythm or paced rhythm as well as sufficient coronary collateral supply, defined as CFI ≥ 0.217 (16).

All original studies were approved by the Ethics Committee of the Canton of Bern, Switzerland, and all patients gave written informed consent for further use of their data.

### Acquisition and manual assessment of the intracoronary electrocardiogram

The acquisition of icECG has been described in detail before (12). In brief, icECG was acquired by attaching an alligator clamp (Stäubli International AG, Pfäffikon, Switzerland) to the 0.014-inch pressure monitoring angioplasty guidewire (PressureWire ™X Guidewire, Abbott, Chicago, IL, United States) positioned in the distal third of a major coronary artery, the clamp was connected to the precordial lead V5. The recording operated at a sampling frequency of 2,000 Hz, and at standard system filtering (Sensis, Siemens Healthineers, Erlangen, Germany; corresponding to a bandpassfilter 0.05–100 Hz and a 22-bit A/D converter). During manual analysis, 12–15 consecutive cardiac cycles were chosen, signal averaged, and, according to the time of recording (i.e., before or at the end of the coronary occlusion), labeled as “non-ischemic” or “ischemic”, finally, resulting in 100 non-ischemic icECGs and 100 ischemic icECGs.

**FIGURE 2:**
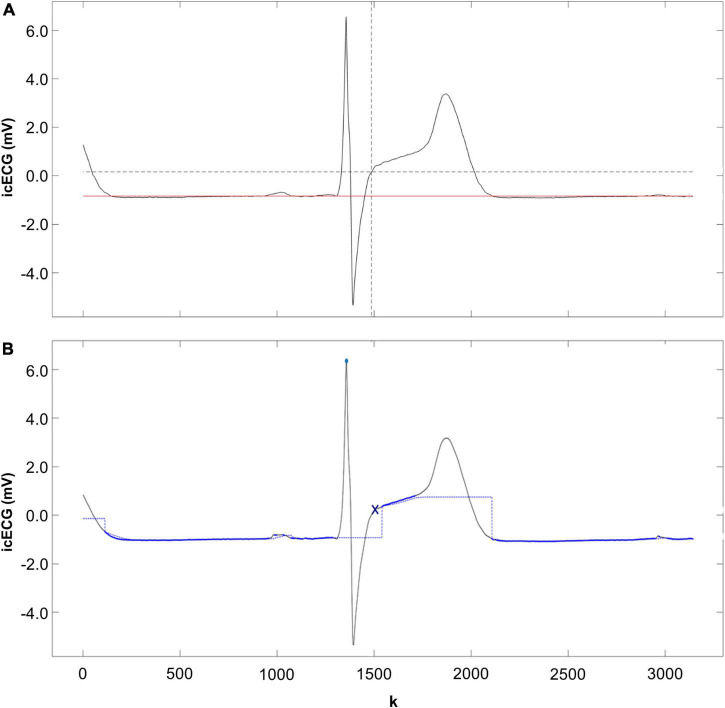
Measurement of the intracoronary electrocardiogram (icECG) ST-segment shift. **(A)** Manually determined icECG ST-segment shift measured at the J-point (intersection between the two dashed black lines). Red line = isoelectric line. **(B)** Autonomous measured icECG ST-segment shift by the newly developed research algorithm. Blue point = R-peak detection, blue line = tracked stable segment of the icECG, representing the isoelectric line before the QRS-complex. Blue x = J-point according to the algorithm. k = number of frames.

Quantitative processing of icECG parameters was performed with customized software (written in Matlab, R2017b), presenting each icECG without information on the ischemic state. For the presented analysis, the results of the manually obtained icECG ST-segment shift (in mV) as obtained by an ECG expert (MRB) in the previous study (15) (second performance) was used (Figure 2A). In brief, measurement of the ST-segment shift was based on the determination of the isoelectric line (Figure 2A, Solid red line) and the Junction-(J)-point (Figure 2A, Intersection between the two dashed black lines). The isoelectric line represents the reference line for the measurement and was set at the PQ-junction, i.e., the end of the PR segment as recommended (17). In case of an atrial repolarization signal (occasionally visible in the icECG), TP-segment served as reference. The J-point was defined as the transition of the QRS-complex to the ST-segment. Using these two cornerstones allowed the calculation of the ST-segment shift as the difference in mV between the isoelectric line and the ST-amplitude at the J-point.

### Autonomous intracoronary electrocardiogram analyzing algorithm

The newly developed algorithm provides a per-beat estimate of the ST-segment shift (Figure 2B), and it works in two steps.

**FIGURE 3:**
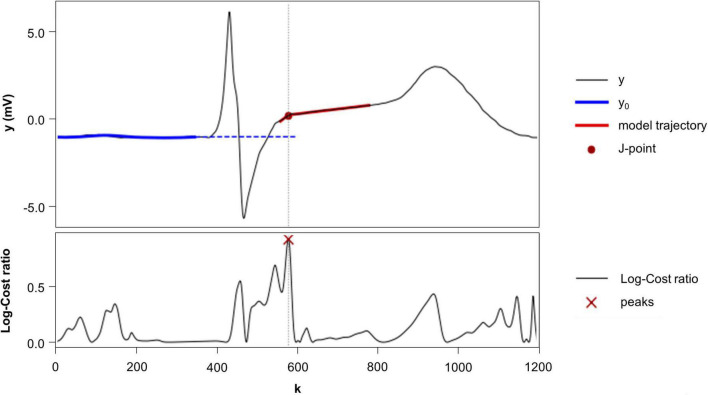
*Upper plot*: Intracoronary electrocardiogram (icECG) signal of a single heartbeat, demonstrating the algorithms operating principle. In a first step, the algorithm reconstructs the isoelectric line (dashed blue line), in a second step the J-point as the transition point between QRS wave and ST-Intervals (red dot). The red lines show the local signal model applied to detect this transition point. Lower Plot: the Log-Cost Ratio (LCR) measure rates the local match of the local signal model to the signal. Note that the LCR tends to peak multiple times around the QRS/T wave: once at around *k* = 450 indicating the S wave, and twice around *k* = 560 and 580 where the J-point is expected; we choose the J point at the later position.

Step 1 reconstructs the icECG signal baseline by defining the isoelectric potential. A moving average filter with a left-sided exponentially decaying window and outlier exclusion is applied onto the signal (18). This leads to a local averaging and extrapolation of the isoelectric potential from the *P*- to the ST-region, where the reference value for the isoelectric value is picked (Figure 3).

Step 2 identifies the point of transition from the QRS-wave to the ST-segment, denoted as the J-point. To this end, a model-based approach with two models is applied, where each model represents an alternative hypothesis: the first model is designed to fit the point of transition (Figure 3) while the second, alternative model, only fits to smooth intervals showing no transitions. Both models are applied in a sliding window manner using the least squares optimum. Finally, the local extrema in the ratio of the cost remainders of the two competitive models are used to identify the point of signal transition, namely, the J-point. This method has been published in detail before (19). Figure 3 shows the application of this method to a single heartbeat.

To ensure low computational complexity and fast processing of the signals, both methods, the moving-average filters, and the transition-point-detection algorithms, are implemented using autonomous linear state space models. These models offer inherent recursive computations and therefore efficient processing (18), thus, providing results before further optimization of the algorithm within 1,000 ms (i.e., within a heartbeat).

### Statistical analysis

The two study groups of non-ischemic and ischemic icECG were based on the two analysing methods, i.e., the results of the ECG expert and the results of the algorithm. Between-group comparison of continuous demographic variables and hemodynamic parameters was calculated by a paired student’s *t*-test.

For determining measurement variability, two way analysis of variance (ANOVA) with two groups (ECG expert and algorithm) and two measurements (non-ischemic, i.e., before coronary occlusion, and controlled ischemic, i.e., at the end of the occlusion), Bland and Altman (20) analysis, as well as intraclass correlation coefficients (ICC) (21) were applied. Inter-method (i.e., inter-rater) ICC was based on an absolute-agreement, two-way random-effects model (*n* = 400). Linear regression analysis was performed for calculation of the regression line in the scatter plots for the illustrative presentation of inter-method variability.

Non-parametric receiver operating characteristics (ROC) curve analysis was used for accuracy assessment of detecting myocardial ischemia by the two analysing methods. Optimal cut-off points for each analysing method were determined by the Youden-Index. Using the DeLong-Test the area under the ROC curves were compared.

Statistical significance was defined at a *p*-level of <0.05. Continuous variables are given as mean ± standard deviation (SD). All analyses were performed using SPSS version 25 (IBM Statistics, Armonk, NY, United States) or MedCalc for Windows, version 19.1 (MedCalc Software, Ostend, Belgium).

## Results

Similar to our previous study (15), two hundred icECGs from 100 patients were included in the study. From each patient, a non-ischemic as well as an ischemic icECG were analyzed. Left anterior descending (LAD) artery served twice as often as the study vessel than the other coronary arteries, namely left circumflex artery (LCX) and right coronary artery (RCA).

### Patient characteristics

Patient characteristics are presented on Table 1.

**TABLE 1:** Patient characteristics.

	Overall
Number of patients	100
**Patient characteristics**	
Age	68 ± 11
Female gender (%)	22
Body mass index (kg/m^2^)	27 ± 4
Angina pectoris before intervention (%)	50
Duration of angina pectoris (months)	11 ± 22
CCS Angina Grade	1.98 ± 0.92
Diabetes mellitus (%)	25
Arterial hypertension (%)	68
Current smoking (%)	14
Cumulative pack years of cigarettes	42 ± 28
Dyslipidemia (%)	76
Family history for CAD (%)	29
Prior myocardial infarction in vessel of interest	10
**Medical treatment**	
Aspirin (%)	84
Platelet inhibitor (%)	41
Calcium channel-blocker (%)	25
Beta-blocker (%)	49
Nitrate (%)	13
Oral anticoagulation (%)	9
Statin (%)	78
ACE inhibitor or ARB (%)	66
Diuretics (%)	31

CCS, Canadian Cardiovascular Society; CAD, coronary artery disease; ACE, angiotensin-converting enzyme; ARB, angiotensin receptor blockers.

### Inter-method variability

**FIGURE 4:**
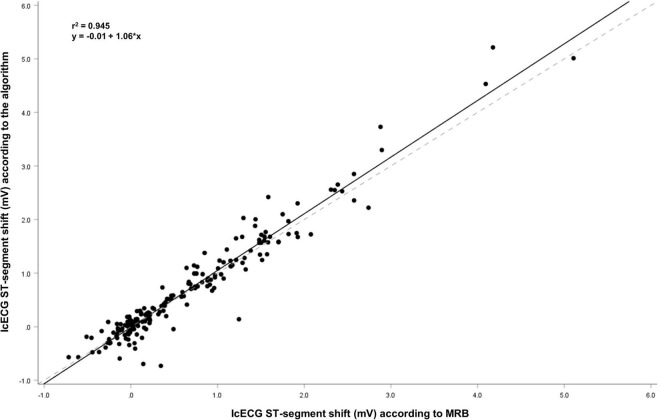
Linear regression between manual and autonomous intracoronary electrocardiogram (icECG) ST-segment shift measurement. Solid black lines = regression lines; dashed gray lines = reference lines, i.e., y = x.

**FIGURE 5:**
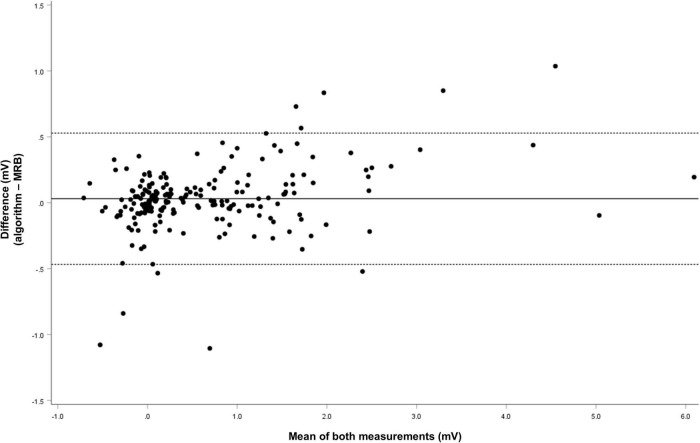
Bland and Altman plots for inter-method variability. Difference = icECG ST-segment shift measured by the algorithm minus manually obtained icECG ST-segment shift; Solid black lines = mean difference; dashed gray lines = 95% limits of agreement. Please see Table 3 for the detailed Bland and Altman analysis.

Two-way ANOVA for repeated measures analysis did not show significant differences between inter-method measurements (Table 2). Determination of the ICC showed the lowest variability for icECG recordings in the LCX (ICC 0.982), followed by the overall analysis (ICC 0.968), the recordings in the LAD (ICC 0.952) and the RCA (ICC 0.931). Please see Table 3 for a presentation of the variability analysis, Figures 4, 5 for graphical illustration of the inter-method variability.

**TABLE 2:** ANOVA.

	Degrees of freedom	Mean square	*F*-value
**Overall, *n* = 200**			
Between groups	1	0.093	0.091;
Within groups	398	1.015	*p* = 0.763

**TABLE 3:** Measurement variability.

		Overall	LAD	LCX	RCA
	Number	200	100	50	50
An error in the conversion from LaTeX to XML has occurred here. 2*Intraclass correlation coefficients	ICC coefficient	0.968	0.952	0.982	0.931
	95% CI	0.958–0.976	0.926–0.968	0.968–0.990	0.881–0.960

An error in the conversion from LaTeX to XML has occurred here. 5*Bland and Altman	Mean_Diff_	0.030	0.071	-0.057	0.037
	SE of Mean_Diff_	0.018	0.025	0.039	0.029
	95% CI for Mean_Diff_	–0.005–0.066	0.021–0.121	–0.133–0.019	–0.021–0.095
	SD_Diff_	0.253	0.254	0.272	0.208
	95% limits of agreement	–0.468–0.528	–0.430–0.572	–0.595–0.480	–0.373–0.447

ICC, intraclass correlation coefficient; CI, confidence interval; LAD, left anterior descending coronary artery; LCX left circumflex coronary artery; RCA, right coronary artery.

### Descriptive statistics

Descriptive statistics of the study parameters are presented on Table 4, grouped according to the respective analysing method. There was no significant difference between the two analysing method in the non-ischemic or the ischemic state in the overall analysis. Further, there was also no significant difference on a per vessel basis.

**TABLE 4:** Study parameters.

	MRB	Algorithm	*p*-value
Overall, *n*	100	100	–
Non-ischemic	0.020 ± 0.247	−0.006 ± 0.269	*p* = 0.475
Ischemic	1.299 ± 0.992	1.386 ± 1.078	*p* = 0.553
**Left anterior descending artery, *n***	**50**	**50**	–
Non-ischemic	0.059 ± 0.214	0.044 ± 0.248	*p* = 0.751
Ischemic	1.219 ± 0.692	1.375 ± 0.832	*p* = 0.719
**Left circumflex artery, *n***	**25**	**25**	–
Non-ischemic	0.022 ± 0.324	−0.093 ± 0.339	*p* = 0.226
Ischemic	2.124 ± 1.264	2.124 ± 1.369	*p* = 0.999
**Right coronary artery, *n***	**25**	**25**	–
Non-ischemic	−0.058 ± 0.208	−0.018 ± 0.212	*p* = 0.504
Ischemic	0.634 ± 0.547	0.668 ± 0.636	*p* = 0.840

### Receiver-operating characteristic curves

**FIGURE 6:**
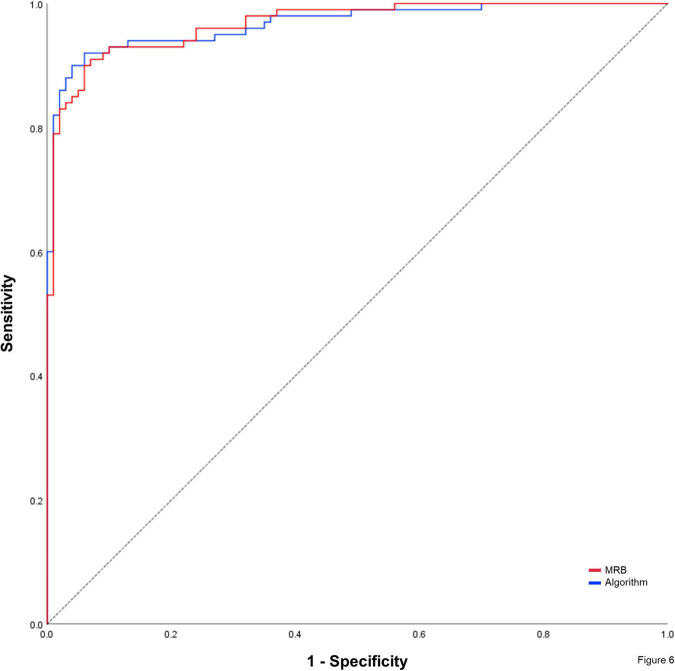
Non-parametric receiver-operating characteristic curve using the time point of intracoronary electrocardiogram (icECG) recording as reference. MRB, manually measured icECG ST-segment shift by an ECG expert. Dashed black line = reference line.

Using the time point of icECG recording as allocation reference for absent or present myocardial ischemia, ROC-analysis of the manually determined icECG ST-segment shift showed an area under the curve (AUC) of 0.968 ± 0.021 (*p* < 0.0001). AUC for icECG ST-segment shift autonomously determined by the algorithm was 0.967 ± 0.023 (*p* < 0.0001; Figure 6). DeLong-Test of the ROC-curves showed no statistically significant difference between the assessment of the ST-segment shift manually or by the algorithm (*p* = 0.925). The same applied for the ROC-analysis on a per vessel basis (Table 5).

**TABLE 5:** Area under the receiver operating characteristics (ROC)-curves.

	MRB	Algorithm	DeLong test
**Overall, *n* = 200**			
Area under the ROC-curve	0.968 ± 0.021	0.967 ± 0.023	*p* = 0.925
**Left anterior descending artery, *n* = 100**			
Area under the ROC-curve	0.979 ± 0.025	0.982 ± 0.025	*p* = 0.630
**Left circumflex artery, *n* = 50**			
Area under the ROC-curve	0.995 ± 0.008	0.994 ± 0.011	*p* = 0.480
**Right coronary artery, *n* = 50**			
Area under the ROC-curve	0.963 ± 0.043	0.931 ± 0.068	*p* = 0.076

Regarding the optimum cut-off for ischemia detection, a manually determined ST-segment shift of 0.358 mV distinguished best between non-ischemic and ischemic ECG, sensitivity 91%, specificity 93%. The best cut-off point for autonomous determined ST-segment shift was 0.318 mV (sensitivity 92%, specificity 94%).

## Discussion

Using an experimental setting with systematically induced, complete coronary balloon occlusion, and thus, absolute myocardial ischemia, non-ischemic and ischemic icECG is distinguishable equally accurate by manual and autonomous quantitative determination of icECG ST-segment shift. In particular, the newly developed algorithm demonstrated an excellent agreement with manual icECG analysis and results available within 1,000 ms. Conversely, manual analysis requires multiple steps cumulating in an analysis time of several minutes.

### Diagnostic value of the intracoronary electrocardiogram

Given the higher sensitivity for myocardial ischemia detection when compared to the surface ECG, icECG has shown ample evidence for its diagnostic value since its first implementation during cardiac catheterization in 1985. In this context, Hishikari et al. (5) assessed the occurrence of ST-segment elevation in the icECG in patients with non-ST-segment elevation myocardial infarction (NSTEMI, diagnosed with the commonly 12-lead ECG) and showed, that 27.6% of patients before percutaneous coronary intervention (PCI) had a significant ST-segment elevation in the icECG (>0.1 mV). This discrepancy between icECG and surface ECG occurred more common in LCX than other culprit lesions (5). In a subsequent clinical trial, the same research group conducted a multiple regression analysis, showing that ST-segment shift in the icECG predicted greater peak values of troponin levels, consistent with greater myocardial injury (8). Thus, they concluded that the icECG “might help to identify high-risk NSTEMI patients with greater myocardial injury” (8).

In patients undergoing elective PCI, Uetani et al. (7) as well as Balian et al. (6) showed that icECG provided a useful method to predict peri-procedural myocardial injury. In this context, Sato et al. (22) have applied the icECG to assess the relation between lipid core burden of coronary lesions (as determined by intravascular ultrasound and near-infrared spectroscopy) and peri-interventional myocardial ischemia by distal embolization of lipid contents. They demonstrated that higher lipid burden was associated with prolonged ST-segment elevation after PCI. Furthermore, Vassilev et al. showed that, after stenting of the main vessel in a bifurcation PCI situation, icECG assessed functionally significant stenosis of the sidebranch with a high diagnostic accuracy (9).

Balian et al. (23) conducted a clinical trial to assess whether icECG ST-segment shift during maximal pharmacologic vasodilation (by intravenous adenosine) could be used as an estimate of hemodynamic relevance of a coronary lesion (as defined by the fractional flow reserve, FFR). They demonstrated a modest linear correlation (*r*^2^ = 0.206) between the tested icECG and the reference FFR and concluded, that icECG “during adenosine infusion may be of value in assessing the functional significance of a borderline stenosis” (23). In a similar trial, using pharmacological inotropic stress induced by dobutamine plus atropine, our research group demonstrated that hyperemic intracoronary ECG ST-segment shift detects structurally relevant coronary stenotic lesions with high sensitivity (12). Hence, we concluded that it could be applied as a high-sensitivity rule out test for relevant stenosis severity.

Due to the icECGs‘ sensitivity to detect changes of myocardial physiology and the fact that myocardial scar remain electrically silent on surface ECG, icECG provides an opportunity to assess myocardial viability during PCI. Petrucci et al. (24) recorded peak-to-peak voltage of the icECG using a common PCI-guidewire electrically isolated by a microcatheter. Subsequently, they matched their results with corresponding segments containing scar, determined by cardiac magnetic resonance imaging as the reference standard. Using this approach, the icECG amplitude (in mV, measured between the highest and the lowest peak of the QRS-complex) distinguished viable from non-viable left ventricular segments with a sensitivity of 99% and a specificity of 69%. Hence, demonstrating the ability of the icECG to provide myocardial viability assessment in real-time during coronary catheterization.

### Research algorithm analysis of intracoronary electrocardiogram

Current manual analysis of the icECG ST-segment shift requires several steps and thus, represents a time-consuming process with personnel effort. As a result, quantitative manual analysis requires at least 5 mins, preventing its application in routine use. In contrast to its application in the icECG, a wide range of methods to analyze ST-segments in 12-lead surface ECG already exists. Many successful approaches use a chain of linear filters and decision rules onto the raw signal and the signals‘ energy. For example, Zong et al. (25) proposes a waveform-length-transformation with a filter applied onto the energy of the signals first derivative. Another common approach, related to the presented approach, is the approximation of the ST-region by a signal model. Firoozabadi et al. (26) successfully applied a polynomial model to the ST-region in order to identify the ST-elevation in an averaged signal.

However, the intracoronary ECG signals has slightly different properties compared to standard surface ECG signals. Because of the direct vicinity to the myocardium, the icECG amplitude is not only several times higher than in the surface ECG, but it records also electrical processes often overseen in the surface ECG (e.g., atrial repolarization or U-waves). Thus, resulting in an unstable baseline (in particular true for the PQ-segment) and P-waves, which can be confused with the QRS-complex. Furthermore, only single-channel measurements (27) are available, preventing derivation of the information from other channels. Last, as the focus is on the progression of the ST-elevation over time, common applied signal averaging prior to analysis, to obtain a noise-reduced signal (27), is not possible. Thus, a conclusive comparison of these methods is not possible, demonstrating the requirement for an icECG-specific analyzing algorithm.

Considering the performance of the algorithm, it demonstrated a low measurement variability compared with the manual analysis performed by an ECG expert. In fact, the algorithm showed a similar inter-rater variability as previously calculated between three different ECG experts on the same data set (inter-rater ICC_*Algorithm*_ 0.968 vs. ICC_*ECGexpert*_ 0.979) (15). Consequently, neither the two-way ANOVA for repeated measures analysis nor the DeLong-Test of the ROC-curves showed significant differences between the two analysing methods in this study. Thus, illustrating the robustness of the novel algorithm.

### Study limitations

Currently, the applied algorithm in this study did not assess the signals real-time, but processed them offline. For a peri-interventional clinically practical diagnostic, online processing and the integration of the algorithm into a full-featured graphical ECG analysis tool would be essential. In foresight, the current method already analyses each heartbeat individually and, with only minor modifications, the required look-ahead can be limited to less than 1,000 ms. Thus, considering a heart rate of 60 bpm (i.e., 1,000 ms per beat), the result can be shown within the next heartbeat.

### Clinical perspectives

As previously demonstrated, the icECG is a sensitive marker for myocardial ischemia enabling multiple applications in daily clinical practice (e.g., coronary stenosis or myocardial viability assessment). However, its implementation requires a robust, real-time system providing autonomously quantitative icECG evaluations. The presented algorithm demonstrates the technical feasibility of a fully autonomous diagnostics tool. As an advantage, our approach operates on single heartbeats, consequently, providing dynamic information on the development and resolution of myocardial ischemia over time (Figure 1C). Analysis of this dynamic behavior, e.g., within the course of a standardized coronary artery balloon occlusion, could provide valuable information about coronary (collateral) flow. However, further studies are necessary to assess the whole potential of icECG in daily clinical practice.

## Conclusion

The newly developed autonomous icECG analysing algorithm is equally accurate as the manual assessment, and consequently, provides the technical fundament for an analysing system to obtain and quantify icECG in real-time.

## Data availability statement

The raw data supporting the conclusions of this article will be made available by the authors, without undue reservation.

## Ethics statement

The studies involving human participants were reviewed and approved by Ethics Committee of the Canton of Bern, Switzerland. The patients/participants provided their written informed consent to participate in this study.

## Author contributions

MB and CS: conception and design, data analysis, interpretation, drafting, and revising of the manuscript. AK-G: data interpretation, drafting, and revising of the manuscript. FW: algorithm development, drafting, and revising of the manuscript. RW: conception and design, algorithm development, interpretation, drafting, and revising of the manuscript. All authors have substantially contributed to the manuscript and approved the submitted version.
